# Limited Phylogeographic Signal in Sex-Linked and Autosomal Loci Despite Geographically, Ecologically, and Phenotypically Concordant Structure of mtDNA Variation in the Holarctic Avian Genus *Eremophila*

**DOI:** 10.1371/journal.pone.0087570

**Published:** 2014-01-30

**Authors:** Sergei V. Drovetski, Marko Raković, Georgy Semenov, Igor V. Fadeev, Yaroslav A. Red’kin

**Affiliations:** 1 Department of Natural History, Tromsø University Museum, University of Tromsø – The Arctic University of Norway, Tromsø, Norway; 2 Department of Vertebrate Zoology, Natural History Museum Belgrade, Belgrade, Serbia; 3 Thematic Group on Bird Ecology, Institute of Systematics and Ecology of Animals of Siberian Branch of Russian Academy of Sciences, Novosibirsk, Russia; 4 Department of Collections, State Darwin Museum, Moscow, Russia; 5 Department of Ornithology, Zoological Museum of Moscow State University, Moscow, Russia; Ben-Gurion University of the Negev, Israel

## Abstract

Phylogeographic studies of Holarctic birds are challenging because they involve vast geographic scale, complex glacial history, extensive phenotypic variation, and heterogeneous taxonomic treatment across countries, all of which require large sample sizes. Knowledge about the quality of phylogeographic information provided by different loci is crucial for study design. We use sequences of one mtDNA gene, one sex-linked intron, and one autosomal intron to elucidate large scale phylogeographic patterns in the Holarctic lark genus *Eremophila*. The mtDNA ND2 gene identified six geographically, ecologically, and phenotypically concordant clades in the Palearctic that diverged in the Early - Middle Pleistocene and suggested paraphyly of the horned lark (*E. alpestris*) with respect to the Temminck's lark (*E. bilopha*). In the Nearctic, ND2 identified five subclades which diverged in the Late Pleistocene. They overlapped geographically and were not concordant phenotypically or ecologically. Nuclear alleles provided little information on geographic structuring of genetic variation in horned larks beyond supporting the monophyly of *Eremophila* and paraphyly of the horned lark. Multilocus species trees based on two nuclear or all three loci provided poor support for haplogroups identified by mtDNA. The node ages calculated using mtDNA were consistent with the available paleontological data, whereas individual nuclear loci and multilocus species trees appeared to underestimate node ages. We argue that mtDNA is capable of discovering independent evolutionary units within avian taxa and can provide a reasonable phylogeographic hypothesis when geographic scale, geologic history, and phenotypic variation in the study system are too complex for proposing reasonable *a priori* hypotheses required for multilocus methods. Finally, we suggest splitting the currently recognized horned lark into five Palearctic and one Nearctic species.

## Introduction

Avise et al. [1] introduced the term "phylogeography" to describe the geographic structuring of mtDNA lineages. A little over a decade later, Avise [2] defined phylogeography as a discipline focused on "the principles and processes governing the geographic distributions of genealogical lineages, especially those within and among closely related species". This discipline is rooted in empirical studies of geographic variation of mtDNA and many phylogeographic studies still use mtDNA markers.

The reasons for the extensive use of mtDNA in phylogeography are well-known: fast substitution rate, lack of recombination, small effective population size resulting in fast lineage sorting and high sensitivity to demographic events. These characteristics, combined with the technical ease of collecting large amounts of sequence data that do not require phasing, make mtDNA the most popular marker for phylogeographic inquiry [2]–[5].

MtDNA has been utilized in a large number of avian phylogeographic studies routinely identifying various degrees of intraspecific lineage sorting or non-random geographic variation of genetic diversity such as geographically concordant clades, isolation-by-distance, geographic clines of genetic diversity, etc. [2], [4].

MtDNA, however, represents only the matrilineal history of avian taxa. Yet, with a few exceptions, females are the dispersing sex in birds - a higher proportion of females disperse from the natal area than males and females disperse over longer distances than males [6]. Thus, maternally inherited mtDNA should be a reliable indicator of phylogeographic patterns among sampled localities.

MtDNA may be susceptible to lineage sorting, branch length stochasticity, and to introgression across taxonomic and biogeographic borders [7]–[10]. The presence of interspecific mtDNA far away from the current hybrid zones has been identified in some avian species [11], but sampling of closely related taxa readily identifies such cases. Furthermore, increasing the sample size and number of characters can also reduce the lineage sorting and branch length stochasticity. This reduction results from sampling of many different haplotypes that increases tree stemminess (the ratio of internal branch lengths to terminal branch lengths) and helps to differentiate the distance between clades from the intraclade variation. Greater stemminess also increases tree resolution and thus, decreases the topological stochastisity [12].

The use of nucleotide sequences from multiple independent nuclear loci is advocated as a better alternative to the use of mtDNA in avian phylogeography [10], [13]. The multi-locus approach widely samples the genome and is predicted to eliminate the lineage sorting and branch length stochasticity, and to increase the resolution of phylogeographic reconstructions [10]. It is also predicted to eliminate the need for sampling of many individuals as long as many loci are sampled [14].

Little empirical evidence, however, supports the utility of multilocus sequence data for avian phylogeography [5]. For example, in a multi-locus study of the red-backed fairy wren (*Malurus melanocephalus*), 35 nuclear loci (almost 15,000 bp/individual) failed to recover phylogeographic structure which was readily recovered with only 467 bp of mtDNA [15]. The authors had to use the phylogeographic structure recovered with their mtDNA data to group individuals for estimating population parameters and levels of gene flow with their nuclear sequences. The isolation with migration analysis [16] based on the mtDNA-guided division produced equal time since divergence between neighboring regions, and the authors again had to resort to mtDNA data to gain "additional perspective on the divergence times" [15].

The red-backed fairy wren study used 30 individuals despite that it focused on taxa with limited geographic variation distributed across a small portion of the smallest continent and the authors emphasized sampling loci over individuals [15]. However, many avian species inhabiting northern continents have very large ranges and population sizes, and exhibit considerable geographic variation. Phylogeographic studies of such taxa demand large sample sizes for sufficient coverage of their ranges and phenotypic variation. Performance of different loci in such challenging conditions becomes important for efficient study design.

In this study we compare the performance of three independent loci (one each of mitochondrial, Z-chromosome linked, and autosomal) individually and of their combined analysis to elucidate the large scale geographic pattern of genetic variation in a small Holarctic genus *Eremophila* (Passeriformes: Alaudidae).

The genus *Eremophila* (Aves: Alaudidae) includes only two currently recognized species - the Temminck's lark (*E. bilopha*) and horned lark (*E. alpestris*). The former species is monotypic and inhabits a narrow belt of rocky deserts following the coastal outline of north Africa and the Middle East from westernmost Mauritania in the west to central Iraq in the east [17]. In contrast, the horned lark is a Holarctic and highly polytypic species with over 40 subspecies [18], [19]. It is the only lark species that has a large Holarctic breeding range whereas the rest of the family is restricted to the Old World. The habitats of the horned lark include arctic and alpine tundra, arid lands with sparse vegetation, and agricultural fields [17], [20].

Nearly two thirds of horned lark subspecies are described from the New World where birds vary primarily in size, back color, and intensity of yellow color on the head, throat, and upper chest [20]. In the Old World, in addition to size and color variation, some alpine subspecies in the central Palearctic are distinguished by a much larger black breast patch which connects with black cheek patches [17]. The remarkable phenotypic variation and large Holarctic breeding range resulted in the original description of many horned lark subspecies, especially those inhabiting the Old World, as distinct species [21].

The taxonomic treatment of the horned lark resembles the taxonomy of the former winter wren (*Troglodytes troglodytes*), which until recently had been treated as a single Holarctic species with over 40 subspecies [18], [19]. However, the survey of the winter wren mtDNA identified a number of deeply divergent, geographically concordant clades in each northern continent [22]. Some of these clades are now recognized as distinct species - the Pacific wren (*Troglodytes pacificus*) in the Pacific Northwest of North America, Winter Wren (*T. hiemalis*) in eastern and northern North America, and the Eurasian wren (*T. troglodytes*) in the Palearctic [23]. Many other Holarctic avian species appear to have divergent Nearctic and Palearctic clades, especially those inhabiting forest habitats [24].

Although the horned lark is not a forest species, recent studies indicate that it may contain deep intra- and intercontinental phylogeographic structuring which remains unrecognized by current taxonomy. A study of the status of the streaked horned lark (*E. a. strigata*) identified three divergent, geographically concordant mtDNA clades just in western North America [25]. Each of these clades included multiple subspecies. These clades formed a monophyletic group with respect to the two Palearctic horned larks used as outgroups, suggesting the possibility of divergence between Nearctic and Palearctic birds. The intercontinental differentiation of horned larks was also supported by a study of divergence between Scandinavian and Nearctic populations of birds based on a fragment of mtDNA CO1 gene [26]. A recent study of the phylogeny of the family Alaudidae found deep divergence among horned lark mtDNA Cytochrome-*b* (cyt-*b*) haplotypes sampled in distant parts of the species range [27]. Furthermore, these divergent haplotypes were paraphyletic with respect to the single cyt-*b* haplotype of the Temminck's lark and Palearctic haplotypes were paraphyletic to Nearctic haplotypes. Therefore, available molecular data argues for the need of a re-evaluation of the species limits within *Eremophila*.

## Materials and Methods

### Ethics Statement

This study did not require ethical approval in our institutions because we used samples loaned to us by public museums or universities (Appendix S1) who comply with relevant regulations for acquisition and curation of their collections.

**Figure 1:**
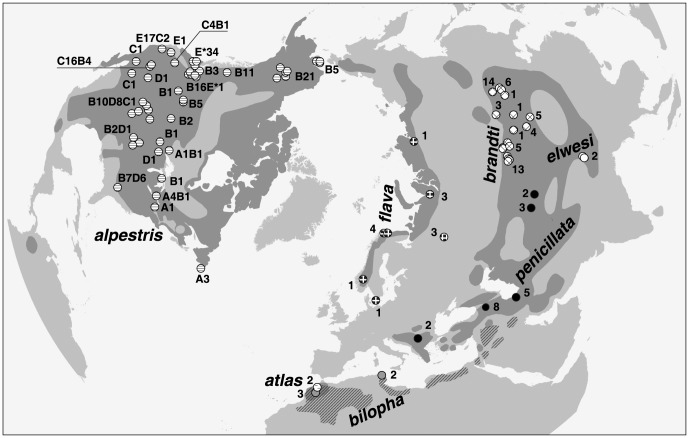
Sampling localities of *Eremophila* larks. MtDNA clades sampled in each locality are identified by clade names from Fig. 2. The numbers following Nearctic clade names indicate their sample sizes. Dark gray areas show ranges of the currently recognized horned lark (solid) and Temminck's lark (striped). The following sources for lark distribution data were used to create this map: [20], [21], [53], [54].

We used a total of 286 horned lark samples, 5 samples of Temminck's lark, and 3 samples of the greater short-toed lark *(Calandrella brachydactyla;* designated as outgroups) obtained from museum collections or academic institutions (Fig. 1; Appendix S1). In our initial Neighbor-Joining analysis of mtDNA ND2 sequences, we tested the following lark genera as potential outgroups: *Alauda, Gallerida, Lullula*, and *Melanocorypha*, but *Callandrella* appeared to be the most closely related to *Eremophila*. This was also confirmed by a recent study of the Alaudidae phylogeny [27].

Most of the samples consisted of muscle tissue preserved in 96% ethanol and have associated voucher specimens in museum collections (Appendix S1). However, the samples from British Columbia, Canada were growing contour feathers and samples from Georgia, USA were amnion harvested from recently hatched eggs (1 per nest). Both the feathers and amnion tissue were preserved in 96% ethanol.

Genomic DNA was extracted using the JETQUICK Tissue DNA Spin Kit (Genomed, Loöhne, Germany) or DNeasy Blood and Tissue Kit (QIAGEN, Valencia, CA, USA) according to the manufacturer’s protocols. We obtained complete mtDNA ND2 gene sequences for 294 of 296 larks sampled for this study. We used 100 ND2 sequences available in GeneBank (accession numbers DQ187388– DQ187487) [25] and two recently published sequences of the streaked horned lark (*E. a. strigata*) [28]. We sequenced 194 additional samples (GeneBank accession numbers: KF735311 - KF735504) using primers and protocols described by Drovetski et al. [29].

We also sequenced intron 9 of the Z chromosome specific Aconitase 1 gene (ACO1I9, 983 bp; GenBank accession numbers: KF735211 - KF735310) for a subset of 59 individuals and autosomal intron 1 of the rhodopsin gene (RHOI1, 922 bp; GenBank accession numbers: KF735505 - KF735628) for a subset of 62 birds. Both subsets included multiple representatives of all ND2 clades. ACO1 was amplified using primers ACO1-I9F2 (CTCCTCTCAGGATCCAGACTT) and ACO1-I9R2 (CAACTTTGTCCTGGGGTCTTT) and annealing temperature 55°C [30]. RHOI1 was amplified using primers RHO-I1F (TGCTACATCGAGGGCTTCTT) and RHO-I1R (CGAGTGACCAGAGAGCGATT) and annealing temperature 56°C [31]. PCR fragments were sequenced in both directions on an ABI 3730 Genetic Analyzer (Applied Biosystems Inc., Foster City, CA). The sequences were aligned automatically in Sequencher 5.0.1 (Gene Codes Corporation, Ann Arbor, MI) and verified manually to ensure consistent alignment of indels.

In heterogametic individuals whose alleles differed in length, the alleles were identified by subtracting the complimentary sequence of the allele without the indel from the double peaks in their chromatogram [32]. Alleles of heterogametic individuals that had the same length but contained multiple nucleotide differences we resolved using PHASE 2.1.1 [33]. We conducted two independent PHASE runs. The first 500 interactions were discarded as burn-in. The following 5000 iterations used a thinning interval of 10.

We used *BEAST 2.0.2 [34] to reconstruct multi-locus species and locus-specific trees and to estimate divergence times among lineages. We used the mean rate of sequence evolution and associated 95% confidence interval (CI) reported by [35] for ND2 (2.9×10^−2^ substitutions/site/Ma [2.4 – 3.3×10^−2^]). For ACO1I9 and RHOI1 we allowed rates to be estimated relative to that of ND2. These estimate were 9.8×10^−3^ substitutions/site/Ma (95% CI: 0.6 – 1.4×10^−2^) and 5.0×10^−3^ substitutions/site/Ma (95% CI: 3.1 – 6.9×10^−3^) respectively.

We used the Bayesian information criterion (BIC) implemented in jModelTest (Posada 2008) to select substitution models for the *BEAST analyses. For ND2 jModelTest selected TrN+G submodel of the generalized time reversible (GTR) model [36] where transversions are weighted equally with discrete-gamma (G) model of substitution rates across sites [37]. For ACO1 jModelTest selected 010220 + I submodel of GTR model with the proportion of invariable sites (I) included. For RHOI1 jModelTest selected K80 [38] with the proportion of invariable sites (I) included (K80 + I). We incorporated a Yule process speciation prior for our *BEAST analysis. To select the appropriate molecular clock prior, we conducted two independent runs for each locus. In one run we used a strict clock prior and in the other relaxed lognormal clock prior. We then conducted a maximum likelihood ratio test [39] to determine whether the strict clock tree likelihood was significantly worse than the relaxed clock tree likelihood. Because MLRT was not significant (all P values > 0.99) for either of our loci, we report the results of our *BEAST analyses with the strict molecular clock prior.

Three separate MCMC analyses were run for 3×10^8^ generations with a 5000 generation burn-in and parameters sampled every 5000 steps. Independent runs were combined using LogCombiner 2.0.2 [34]. Tracer 1.5 (http://beast.bio.ed.ac.uk/Tracer) was used to determine the effective sample size of each parameter and calculate its mean and 95% highest posterior density (95% HPD) interval. Tree topologies were assessed using TreeAnnotator 2.0.2 [34] and visualized in FigTree 1.3.1 (http://tree.bio.ed.ac.uk/software/figtree/).

We used TCS 1.21 [40] to reconstruct allele networks for nuclear loci. Indels were treated as missing data.

## Results

### Phylogeny of mtDNA haplotypes

**Figure 2:**
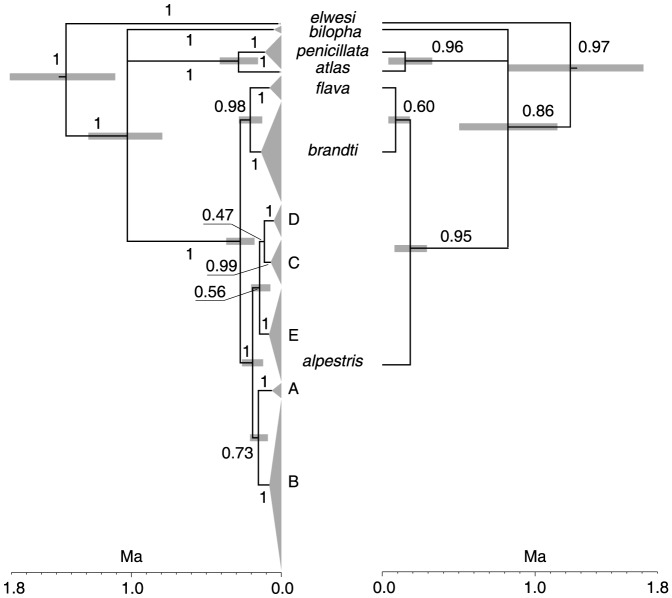
Phylogenetic tree of mtDNA ND2 haplotypes (left) and the species tree based on ND2 sequences (right). Palearctic clades are identified by subspecific names. Nearctic clades are identified by letters (A - E) due to overlap of their ranges. Numbers next to branches show their posterior probability. Gray bars next to nodes indicate their 95% HPD interval for the node age. Scale below each tree indicates time in million years (Ma).

Monophyly of both *Calandrella* and *Eremophila* was strongly supported in the phylogenetic analysis of ND2 haplotypes. Both posterior probability values (PP) were equal to 1. The divergence date between these genera was estimated at the Pliocene, 4.9 Ma (95% HPD 3.7–6.4 Ma). *Eremophila* consisted of 11 strongly supported clades (all PPs ≥ 0.99; Fig. 2) diversification of which began with the divergence of Tibetan horned larks (*elwesi*; Fig. 1) from all other *Eremophila* in the Early Pleistocene, 1.4 Ma (1.1–1.8 Ma). The initial divergence of *elwesi* from all other *Eremophia* was followed by a trichotomous split into *bilopha*, south Palearctic alpine (*atlas* and *penicillata* on Fig. 1), and other Palearctic (*flava* and *brandti*) and Nearctic (*alpestris*) clades. This split occurred at the end of the Early Pleistocene, 1 Ma (0.8 – 1.3 Ma). The closer relationship of *bilopha* to all other horned larks than *elwesi* makes currently recognized horned lark paraphyletic in respect to the Temminck's lark.

**Figure 3:**
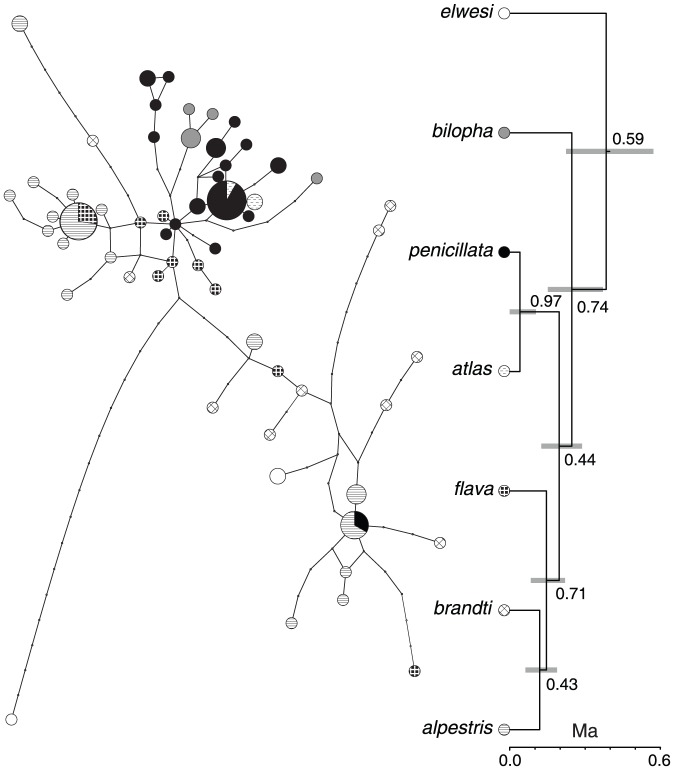
An allele network and species tree based on ACO1I9 sequences. MtDNA clades are identified by names from Fig. 2. Numbers next to branches show their posterior probability. Gray bars next to nodes indicate their 95% HPD interval for the node age. Scale below each tree indicates time in million years (Ma).

**Figure 4:**
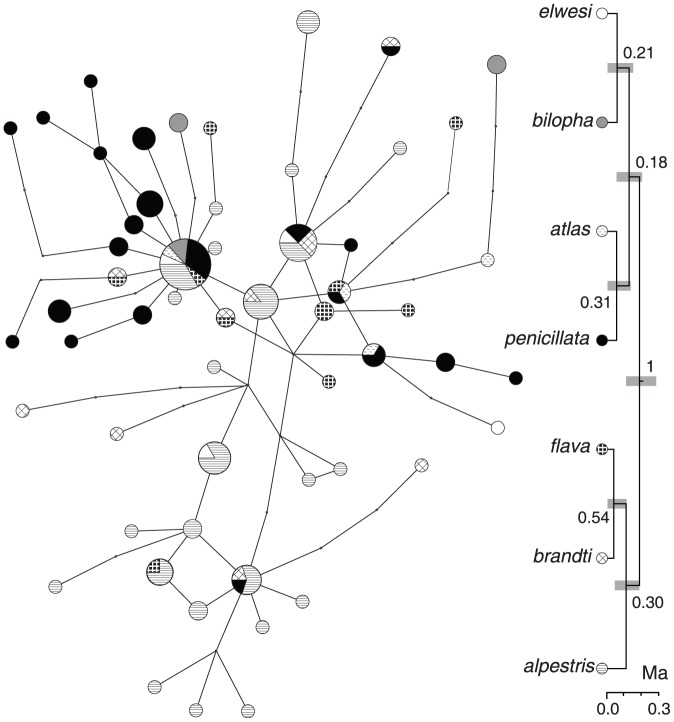
An allele network and species tree based on RHOI1 sequences. MtDNA clades are identified by names from Fig. 2. Numbers next to branches show their posterior probability. Gray bars next to nodes indicate their 95% HPD interval for the node age. Scale below each tree indicates time in million years (Ma).

**Figure 5:**
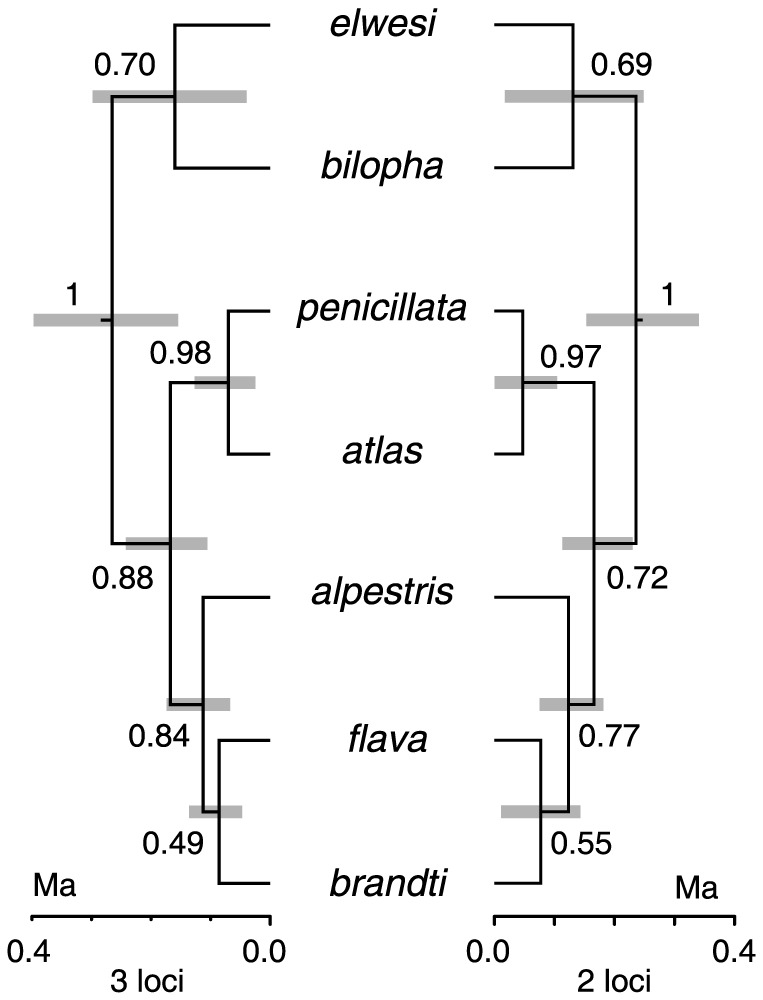
Species trees based on all 3 loci (left) and two nuclear loci (right). Numbers next to branches show their posterior probability. Gray bars next to nodes indicate their 95% HPD interval for the node age. Scale below each tree indicates time in million years (Ma).

All Palearctic clades were geographically concordant (Fig. 1). In the addition to *elwesi* and *bilopha*, two Moroccan alpine horned larks (*atlas*) formed a clade which was the sister to a clade composed of west and central Palearctic alpine larks (*balcanica*, *penicillata*, and *albigula*). The second subspecies has a priority over the other two, so we refer to this clade as *penicillata* (Figs. 1, 2, 3, 4, 5). The *atlas* and *penicillata* clades diverged in the Middle Pleistocene 0.286 Ma (0.155–0.410 Ma). The earliest paleontological records for the horned lark from the range of *pennicillata* in the Caucasus are dated at 0.393 ± 0.027 Ma [41].

The last two sister Palearctic clades, *flava* and *brandti* (Fig. 2), were more closely related to Nearctic horned larks than to other Palearctic clades. One of these clades was comprised of north Palearctic birds (*flava*) and the other of birds inhabiting central Palearctic aridlands (*brandti*; Fig. 1). The earliest paleontological records of the horned lark from Europe (southern France and southeastern Germany) that is likely to represent *flava* date to 0.42 ± 0.05 Ma and from northeastern China (likely *brandti*) date to 0.2 – 0.6 Ma.

Divergence of *flava* and *brandti* and of their common ancestor from Nearctic larks dated to the Middle Pleistocene, 0.208 Ma (0.128 – 0.283 Ma) and 0.274 Ma (0.179 – 0.367 Ma), respectively. All Nearctic horned larks were monophyletic (PP  =  1; Fig. 2), suggesting a single colonization of the Nearctic by horned larks at the end of Middle Pleistocene, between 0.179 and 0.367 Ma. The horned lark has been recorded in the Bartek Quarry in eastern Nebraska, USA dating to the Middle Pleistocene [42]. Therefore, our estimate of the time of colonization of the Nearctic by the horned lark agrees with the available paleontological data.

Despite a relatively recent history of the horned lark in the Nearctic, the number of strongly supported clades with PP ≥ 0.99 (5) was similar to that in the Palearctic (6). However, none of the three internal nodes connecting Nearctic clades had statistical support (PP ≤ 0.72). Four of the five Nearctic clades had relatively well defined geographic ranges with only limited overlap (Fig 1).

The range of the clade E extended across the Pacific USA from western Washington through western California. All 32 individuals of streaked horned lark (*E. a. stigata*) from western Washington and two from Portland, Oregon shared the same unique ND2 haplotype and are identified by "E*" in Fig. 1. Only a single bird carrying the E* haplotype was found among 17 larks sampled in eastern Washington.

Clade C can be characterized as a Great Basin and southwestern US clade (Fig. 1). C-clade haplotypes dominated samples from south-central Oregon and Nevada. The single samples from southernmost California and central Arizona also belong to this clade. Two of the 19 birds sampled in the Central Valley of California had C-clade haplotypes as well as one bird collected in the Rocky Mountains of central Colorado.

Clade D was widespread across the central part of the contiguous USA from Utah to Wisconsin and Georgia (Fig. 1) and clade A had northeastern Nearctic range and was recorded in Minnesota and New York, USA and Ontario and Newfoundland Is., Canada.

In contrast to other four Nearctic clades, clade B had the largest range and overlapped substantially with all other clades (Fig. 1). Its range extended from northwestern Alaska to Nevada in the southwest, to Georgia in the southeast, and Ontario to the northeast. It was the only clade found among our samples from British Columbia, Canada and from Alaska, alpine and eastern Washington (except a single E* haplotype), Montana, North Dakota, and Minnesota, USA.

Due to extensive geographic overlap of the clade B range with ranges of other Nearctic clades and the monophyly of all Nearctic clades, in the species tree reconstruction we combined all Nearctic birds into a single group identified as *alpestris* for this name has priority among all Nearctic subspecies of the horned lark. The species tree based on ND2 sequences had the same topology and very similar, although slightly younger date estimates for the divergence events (Fig. 2). The biggest difference between ND2 haplotype and species trees was lower PP for all nodes. For two nodes, monophyly of *bilopha* with (*penicillata*, atlas) and ((*flava*, *brandti*), *aplestris*) in respect to *elwesi* and monophyly of *flava* with *brandti* in respect to *aplestris*, the PP probabilities fell below the conventional 0.95 statistical significance cut-off value, from 1 to 0.86 and from 0.98 to 0.6 respectively.

### Phylogeny of nuclear haplotypes

Trees based on alleles of nuclear loci strongly supported monophyly of *Eremophila* and *Calandrella* (PP ≥ 0.98). The divergence date between the two lark genera was estimated at 2.1 Ma (1.5–2.8 Ma) for ACO1I9 and 2.8 Ma (1.8 – 4.8 Ma) for RHOI1. These estimates were significantly lower than the estimate of 4.9 Ma (95% HPD 3.7–6.4 Ma) based on ND2 sequences.

Genetic variation within *Eremophila* appeared poorly structured in both nuclear loci trees (Figs. 3 and 4). Despite the lack of structure, most of ACO1I9 alleles were unique to mtDNA clades. Only three ACO1I9 haplotypes were shared by individuals with mtDNA from different clades. Two of these alleles were shared by individuals with mtDNA from closely related clades (*penicillata* and *atlas*; *alpestris* and *flava*) and one was shared by individuals with distantly related mtDNA clades (*apestris* and *pennicillata* Fig. 3) from distant geographic areas: Kazakhstan, Washington, and Colorado.

In contrast, many RHOI1 alleles were shared by larks carrying mtDNA haplotypes from multiple clades (Fig. 4). The most common RHOI1 allele was shared by individuals from six mtDNA clades on three continents, and another five alleles were found on both northern continents.

The lack of lineage sorting and sharing of alleles had a profound effect on the depth and resolution of the species tree based on nuclear loci. Although the topology of the ACO1I9 species tree was almost identical to that of the ND2 tree (the only exception was the sister relationship of *brandti* and *alpestris* instead of *brandti* and *flava*), only a single node (*penicillata* + *atlas*) had PP > 0.95 whereas all other nodes, including the monophyly of *Eremophila*, had PP ≤ 0.74 (Fig. 3). The divergence of *Calandrella* and *Eremophila* was dated to 0.522 Ma (0.214 – 1.675 Ma) and root of *Eremophila* to 0.384 Ma (0.224 – 0.572 Ma). The split between Nearctic *alpestris* and its Palearctic sister *brandti* was dated to 0.120 Ma (0.063 – 0.189 Ma).

In the RHOI1 species tree, the monophyly of *Eremophila* was strongly supported (PP  =  1) whereas all other nodes had very low PP values (0.18 ≤ PP ≤ 0.54, Fig. 4). The topology of this tree was similar to that of the ND2 species tree, except *bilopha* appeared to be the sister of *elwesi*, rather than to other horned larks. The divergence of *Calandrella* and *Eremophila* was dated to 0.623 Ma (0.276 – 1.145 Ma) and root of the *Eremophila* to 0.181 Ma (0.105 – 0.276 Ma). The split between Nearctic *alpestris* and its Palearctic sister *brandti* was dated to 0.106 Ma (0.041 – 0.180 Ma).

### Multilocus reconstruction of the species tree

The topologies and node support of the species trees based on all three loci and on two nuclear loci were very similar (Fig. 5). The topology of both trees was the same as the topology of the species tree based on RHOI1 (Fig. 4) and differed from ND2 tree topology by positioning *bilopha* as the sister to *elwesi*. Although nodes in the multilocus species trees were much better supported than in the RHOI1 tree, the PP values were > 0.95 only for the monophyly of *Eremophila* and sister relationship between *penicillata* and *atlas*.

Despite the similarity of topologies among single locus and multilocus species trees, the divergence date estimates differed significantly (Table 1). Divergence date estimates were oldest for the ND2 haplotype tree followed closely by the ND2 species tree, whereas the estimates for individual nuclear loci species trees and multilocus species trees were several fold lower.

**Table 1:** Divergence time estimates and their 95% HPD intervals for selected nodes.

Tree	Date, Ma	Low 95% HPD	High 95% HPD	% of the mean
**split of *Eremophila* and *Calandrella***				
ND2 haplotypes	4.936	3.673	6.403	55%
ND2 species	4.528	1.209	6.309	113%
ACO1I9	0.522	0.214	1.675	280%
RHOI1	0.623	0.276	1.145	139%
2 nuclear loci	0.985	0.403	1.961	158%
3 loci	1.674	0.709	2.974	135%
**node: root of *Eremophila***				
ND2 haplotypes	1.436	1.108	1.812	49%
ND2	1.257	0.826	1.709	70%
ACO1I9	0.384	0.224	0.572	91%
RHOI1	0.181	0.105	0.276	94%
2 nuclear loci	0.236	0.153	0.341	80%
3 loci	0.266	0.155	0.397	91%
**divergence of *alpestris* from Palearctic birds**				
ND2 haplotypes	0.274	0.179	0.367	69%
ND2	0.186	0.08	0.291	113%
ACO1I9	0.12	0.063	0.189	105%
RHOI1	0.106	0.041	0.18	131%
2 nuclear loci	0.123	0.075	0.181	86%
3 loci	0.113	0.067	0.174	95%
**split of *atlas* and *penicillata***				
ND2 haplotypes	0.286	0.155	0.41	89%
ND2	0.152	0.04	0.327	189%
ACO1I9	0.041	0	0.105	256%
RHOI1	0.052	0	0.131	252%
2 nuclear loci	0.047	0	0.105	223%
3 loci	0.07	0.025	0.127	146%

The comparison of the divergence date estimates of different trees with the available paleontological data suggest that the nuclear species trees and multilocus species trees significantly underestimate divergence dates. For example, the oldest known records of the horned lark from Caucasus are 0.393 ± 0.027 Ma, Europe – 0.420 ± 0.050 Ma, northeastern China – 0.2 – 0.6 Ma [41], and from Nebraska - Middle Pleistocene [42] which corresponds to 0.126 – 0.781 Ma. According to the estimates of the 3-loci species tree, the initial divergence within Eremophila is younger than the three Palearctic records listed above. Furthermore, only the higher 95% HPD interval limit (0.178 Ma) of the divergence between *alpestris* and the common ancestor of *flava* and *brandti* falls within the Middle Pleistocene, whereas the mean (0.113 Ma) falls within the Late Pleistocene.

In contrast to multilocus species tree, the ND2 haplotype tree produced divergence date estimates consistent with available paleontological data. The earliest record of the horned lark from the Caucasus (0.393 ± 0.027 Ma) is older than the estimate of the divergence date between *pennicillata* and *atlas* 0.286 Ma (95% HPD 0.155 – 0.410 Ma) but younger than the divergence date estimate for the split of the common ancestor of *pennicillata* and *atlas* from other horned lark clades (1.041 Ma; 0.794 – 1.289 Ma). The oldest records from Europe (0.420 ± 0.050 Ma) and northeastern China (0.2 – 0.6 Ma) fall between the *branti*/*flava* split (0.208 Ma; 0.128 – 0.283 Ma) and divergence of their common ancestor from other horned larks (1.041 Ma; 0.794 – 1.289 Ma). Finally, the divergence of *alpestris* from the common ancestor of *branti* and *flava* 0.274 Ma and its entire 95% HPD interval (0.179 – 0.376 Ma) falls within the Middle Pleistocene (0.126 – 0.781 Ma), the period to which the earliest Nearctic record of the horned lark belongs.

The node ages estimated using our ND2 haplotype and multilocus datasets were strongly correlated. This correlation was not linear and fit the shifted power model: multilocus date  =  0.005332 × (ND2 date + 2.372115)^2.89062^; df  =  3, r^2^  =  0.9993, P < 0.0001. Therefore, the difference in divergence date estimates between mtDNA and multilocus data is greatest at the most recent dates then slowly decreases towards the older dates. According to this relationship, both trees will converge to similar date estimates for divergences older than 12 Ma.

## Discussion

### Performance of individual loci and multilocus analysis

We used one mtDNA, one Z-linked, and one autosomal locus to identify the pattern of geographic structuring of genetic variation within a small lark genus *Eremophila* that consists of two currently recognized species. All three loci individually and their joint analysis support the monophyly of the genus and paraphyly of the horned lark in respect to the Temminck's lark. However, in the reconstruction of the relationships within *Eremophila* the performance of the loci differed significantly.

The tree based on mtDNA ND2 gene identified 11 strongly supported and geographically concordant clades - 6 in the Palearctic and 5 in the Nearctic (Fig. 2). Only Nearctic clades which diversified at the end of the Middle - Late Pleistocene were partially overlapped geographically and relationships among them were weakly supported. Older Palearctic clades did not overlap except, perhaps, *bilopha* and *atlas* in Morocco, where they prefer different habitats (alpine versus rocky deserts, respectively). The relationships among Palearctic clades were well supported with only a single trichotomous split.

The structuring of Palearctic mtDNA clades was not restricted to geography. Old World clades differed in ecology and plumage patterns. These clades could be characterized as inhabiting rocky deserts (*bilopha*), Asian aridlands with sparse grassy and shrubby vegetation (*brandti*), arctic tundra (*flava*), and alpine habitats (*elwesi*, *penicillata*, and *atlas*). Among the latter, *pennicillata* whose range is situated between ranges of distantly related *elwesi* and the closely related sister clade *atlas*, has the most distinct among all *Eremophila* plumage pattern. The black color on the face and upper chest of *penicillata* occupies a much larger area than in other horned larks and is connected, leaving only a small light patch on the throat, whereas in all other forms the black color is not connected and forms separate cheek patches and a bib.

In contrast to mtDNA, nuclear loci provided limited information on structuring of genetic variation within *Eremophila* and provided poor support for the clades identified by mtDNA ND2 sequences. The presence of mtDNA structure concordant with geography, ecology, and plumage patterns and its lack in nuclear or multilocus trees should not be interpreted as disagreement between mtDNA and nuclear loci resulting from the stochastic nature of the coalescence or male-biased dispersal [4], [43], [44]. Due to differences in the effective population size (N_e_) of mtDNA and nuclear loci, the lineage sorting of mtDNA haplotypes requires a quarter of time needed for autosomal loci or a third relative to Z-linked loci. Indeed, the shifted power relationship between divergence date estimates based on ND2 and multi-locus species trees, suggest that for a long time, perhaps as long as 12 Ma, nuclear loci appear to underestimate the age of the events relative to the mtDNA. On the other hand, date estimates based on mtDNA appear to correspond well with the available paleontological data.

In a recent study of rosyfinches (Fringillidae: *Leucosticte*), ACO1I9 and autosomal melanocortin 1 receptor gene (MC1R) failed to identify structuring among species that had a similar level of divergence in the ND2 tree to that of *Eremophila* clades. Species monophyly in *Leucosticte* was strongly supported by maximum likelihood bootstrap values in the mtDNA ND2 tree [44]. In another recent study of the phylogeny of accentors (*Prunellidae*), ACO1I9 performed well identifying lineages that diverged 6 – 3 Ma ago, however, the relationships among lineages younger than 2.5 Ma were unresolved and some species appeared paraphyletic [32]. These and our current findings suggest that nuclear loci may be of limited utility for phylogeographic studies dealing with lineages evolved in the Middle or Late Pleistocene because they are indeed lagging indicators of divergence events [4].

Combining nuclear loci into a single phylogenetic analysis did not improve phylogenetic resolution. Our multilocus species tree topology was virtually identical to that of the ND2 tree. The only difference was the position of *bilopha* as the sister to *elwesi* in the former and as the sister to all other horned larks in the latter tree. However, only a single of five nodes had statistical support in the species tree, whereas in the ND2 tree, four of these nodes were strongly supported. A number of recent studies using mtDNA and nuclear loci reported similar results - nuclear loci identify phylogeographic structure similar to mtDNA but with poor statistical support if the number of loci is small and increasing with number of loci sampled regardless of whether population divergence was relatively deep [24], [32], [44]–[49] or shallow [48], [50], [51].

Furthermore, our species tree had nearly twice the 95% HPD intervals for node ages relative to their mean value than our ND2 haplotype tree. For the two nodes that had statistical support (PP ≥ 0.98) in both trees, the ancestral node of *Eremophila* and the node connecting *atlas* and *penicillata*, the intervals were 91% and 146% of the mean in the species tree but only 49% and 89% respectively in the ND2 tree. Perhaps, if the group membership is known and a large enough number of nuclear loci is used, they may resolve phylogeographic structure more precisely than a single mtDNA locus.

### Systematics implications

All three loci and the multilocus species tree identified paraphyly of the horned lark in respect to the Temminck's lark and therefore, suggest the need for taxonomic revision of *Eremophila*. However, due to the lack of nodal support in our multilocus species tree, we discuss the taxonomic implications of our study in light of the relationships among mtDNA clades identified in our ND2 tree (Fig. 2). Although matrilineal history may not be completely representative of the evolutionary history of *Eremophila* and the relationships among evolutionary units within it, for reasons discussed in the Introduction, we believe that mtDNA is capable of identification of the presence of such units. Furthermore, our estimates of node ages based on the ND2 trees appear to fit the available paleontological data.

According to our mtDNA ND2 tree, the first clade to diverge from the other *Eremophila* was *elwesi*. This clade was composed of only two samples from Tibet in our tree, but it is likely that several other subspecies from central China and Himalayas may belong to this clade as well. For example, in the mtDNA cytochrome-*b* gene tree horned larks from Pakistan were closely related to *elwesi* samples [27].

The initial split of *elwesi* was followed by a trichotomy of *bilopha*, *atlas* + *penicillata*, and the lineage that included *flava*, *brandti*, and all Nearctic larks. The Temminck's lark is already recognized as a distinct species that inhabits rocky deserts inland from the cost of North Africa, Arabia, and Middle East (Fig. 1). The second major lineage combines two closely related, sister clades of high alpine larks. One clade, *atlas*, corresponds to the currently recognized subspecies *E. a. atlas* inhabiting Atlas mountains of Morocco. Its sister clade, *penicillata*, includes birds inhabiting alpine zone of Palearctic mountains from southeastern Europe to Tian-Shan. In our study this clade was comprised of three subspecies: *E. a. balcanica*, *E. a. penicillata*, and *E. a. albigula*. All these subspecies have black color on the face, neck, and the bib connected, whereas all other larks have a clear separation of the black color between the bib and neck.

The third major, Holarctic lineage was divided into two sister Palearctic and five closely related Nearctic clades. The two Palearctic clades corresponded to subspecies *E. a. flava* breeding in the tundra from Scandinavia to Amguema River in Chukotka and *E. a. brandti* breeding in aridlands from the northwestern border of Kazakhstan to northeastern Inner Mongolia in China.

Although the monophyly of Nearctic clades was strongly supported, the relationships among them were unresolved. Furthermore, breeding range of one clade (B) overlapped with ranges of other clades. Much more detailed sampling of Nearctic horned larks is required to reconstruct clade ranges and elucidate their evolutionary history.

Therefore, our data suggest that all Palearctic clades represent independent evolutionary units and should be treated as distinct species: *elwesi* (although *longirostris* should have priority if it belongs to this clade), *bilopha*, *atlas*, *penicillata*, *brandti*, and *flava*. Nearctic populations should be treated as a single species - *alpestris* pending further investigation.

## Conclusions

Phylogeographic studies of Holarctic taxa are challenging due to sample sizes and geographic coverage required for deciphering of even large scale patterns of genetic variation. This is especially true for abundant, wide ranging taxa like *Eremophila* that have extensive phenotypic and ecological variation. Such extensive variation (> 40 described subspecies) prevents a meaningful *a priori* designation of evolutionary units required for analyses of multiple loci with extensive incomplete sorting. Our data show that mtDNA is capable of identifying geographic patterns of genetic variation within and among closely related avian taxa and, ultimately, independent evolutionary units. This power comes from several fold faster lineage sorting and inheritance through females - the further and more frequently dispersing sex in most birds. Our data also show that the intrataxon geographic pattern of mtDNA variation is correlated with the ecological and phenotypic variation and should not be readily dismissed as the result of stochasticity of demographic and evolutionary processes. Conversely, nuclear loci may provide little resolution for the identification of patterns of genetic variation and evolutionary units within abundant, wide-ranging taxa. Perhaps, due to their large effective population size, the lineage sorting of nuclear alleles cannot be completed or significantly advanced between the consecutive recent divergence events. The current study and others, e.g. *Leucosticte* [44], are cases in point. On the other hand, divergent Nearctic lineages within the winter wren identified using only mtDNA [22] were later confirmed to be reproductively isolated [52] and ultimately recognized as distinct species [23].

Thus, we suggest that phylogeographic studies of wide-ranging, abundant birds should place an emphasis on first exploring the geographic variation in mtDNA by sampling a large number of individuals from as many geographic localities as possible. Once this pattern is established, variation in at least a modest number of nuclear and especially Z-linked loci should be explored.

## Supporting Information

Appendix S1 **Samples used in this study and GenBank accession numbers.**
(PDF)
