# Supplementary material for: Limited Phylogeographic Signal in Sex-Linked and Autosomal Loci Despite Geographically, Ecologically, and Phenotypically Concordant Structure of mtDNA Variation in the Holarctic Avian Genus Eremophila
Source: PLoS One. 2014 Jan 30;9(1):e87570. doi: 10.1371/journal.pone.0087570 (PMC3907499; doi:10.1371/journal.pone.0087570)
Supplement: Appendix S1 — Samples used in this study and GenBank accession numbers. (PDF) [file pone.0087570.s001.pdf]

Appendix S1. Samples used in this study and GenBank accession numbers

ID	Institution	Species	mtDNA clade	Sex	Date	Lat.	Lon.	ND2	ACO1	RHO
SVD2112	UWBM 64797	*Calandrella brachydactyla*	*Calandrella*	M	14-Jul-99	43.90	40.78	KF735312	KF735211, KF735212	KF735505, KF735506
CDS4814	UWBM 46251	*Calandrella brachydactyla*	*Calandrella*	F	19-May-93	43.83	76.35	KF735311		
SVD1138	UWBM 56909	*Calandrella brachydactyla*	*Calandrella*	F	2-Jun-96	46.22	47.77	KF735313	KF735213	KF735507, KF735508
NHMUO17026	NHMUO 17026	*Eremophila alpestris*	*elwesi*		15-Jul-06	30.26	90.66	KF735315	KF735214, KF735215	KF735509, KF735510
NHMUO17038	NHMUO 17038	*Eremophila alpestris*	*elwesi*		14-Jul-06	30.74	90.95	KF735314	KF735216	KF735511, KF735512
AGB1	U. de Montpellier	*Eremophila bilopha*	*bilopha*	M		34.03	353.15	KF735318	KF735217, KF735218	KF735527, KF735528
AGB2	U. de Montpellier	*Eremophila bilopha*	*bilopha*	M		34.03	353.15	KF735319	KF735219, KF735220	KF735531, KF735532
AGB3	U. de Montpellier	*Eremophila bilopha*	*bilopha*			34.03	353.15	KF735320	KF735221	KF735533, KF735534
ZMUC135714	ZMUC 135714	*Eremophila bilopha*	*bilopha*			36.78	10.17	KF735316		
ZMUC135715	ZMUC 135715	*Eremophila bilopha*	*bilopha*			36.78	10.17	KF735317	KF735222	
AGA1	U. de Montpellier	*Eremophila alpestris*	*atlas*	M		34.03	353.15	KF735321	KF735223, KF735224	KF735525, KF735526
AGA2	U. de Montpellier	*Eremophila alpestris*	*atlas*			34.03	353.15	KF735322	KF735225	KF735529, KF735530
MR1061	NHMB	*Eremophila alpestris*	*penicillata*	M	5-Aug-10	43.31	22.86	KF735442	KF735226, KF735227	KF735583, KF735584
MR1062	NHMB	*Eremophila alpestris*	*penicillata*	M	5-Aug-10	43.31	22.86	KF735443	KF735228, KF735229	KF735585, KF735586
MR0950	NHMB	*Eremophila alpestris*	*penicillata*	M	18-Jun-10	40.47	44.19	KF735444	KF735230, KF735231	KF735575, KF735576
MR0951	NHMB	*Eremophila alpestris*	*penicillata*	M	18-Jun-10	40.47	44.19	KF735445	KF735232, KF735233	KF735577, KF735578
MR0952	NHMB	*Eremophila alpestris*	*penicillata*	M	18-Jun-10	40.47	44.19	KF735446	KF735234, KF735235	KF735579, KF735580
MR0953	NHMB	*Eremophila alpestris*	*penicillata*	M	18-Jun-10	40.47	44.19	KF735447	KF735236, KF735237	KF735581, KF735582
IVF1021	SDM	*Eremophila alpestris*	*penicillata*	M	25-May-11	40.43	44.23	KF735331	KF735238, KF735239	KF735571, KF735572
IVF1100	SDM	*Eremophila alpestris*	*penicillata*	F	16-Jun-11	40.42	44.25	KF735332	KF735240	KF735573, KF735574
SVD4632	SDM	*Eremophila alpestris*	*penicillata*	M	25-May-11	40.43	44.23	KF735486	KF735241, KF735242	KF735623, KF735624
SVD4633	SDM	*Eremophila alpestris*	*penicillata*	M	25-May-11	40.43	44.23	KF735487	KF735243, KF735244	KF735625, KF735626
EAK192	MSUZM	*Eremophila alpestris*	*penicillata*	M	18-May-05	35.99	51.63	KF735323	KF735245, KF735246	KF735543, KF735544
EAK193	MSUZM	*Eremophila alpestris*	*penicillata*	M	18-May-05	35.99	51.63	KF735324	KF735247, KF735248	KF735545, KF735546
EAK194	MSUZM	*Eremophila alpestris*	*penicillata*	M	18-May-05	35.99	51.63	KF735325	KF735249, KF735250	KF735547, KF735548
EAK195	MSUZM	*Eremophila alpestris*	*penicillata*	M	18-May-05	35.99	51.63	KF735326		KF735549, KF735550
EAK196	MSUZM	*Eremophila alpestris*	*penicillata*	M	18-May-05	35.99	51.63	KF735327		KF735551, KF735552
GAV167	UWBM 46543	*Eremophila alpestris*	*penicillata*	M	24-May-93	42.98	75.88	KF735328	KF735251, KF735252	KF735555, KF735556
GAV168	UWBM 46544	*Eremophila alpestris*	*penicillata*	F	24-May-93	42.98	75.88	KF735329		
GAV169	UWBM 46545	*Eremophila alpestris*	*penicillata*	M	24-May-93	42.98	75.88	KF735330	KF735253, KF735254	KF735557, KF735558
F091	MSUZM	*Eremophila alpestris*	*penicillata*	M	11-May-10	42.95	80.00		KF735255, KF735256	KF735553, KF735554
IVYBN	MSUZM	*Eremophila alpestris*	*penicillata*	M	11-May-10	42.95	80.00	KF735333	KF735257, KF735258	KF735521, KF735522
NHMUO18830	NHMUO 18830	*Eremophila alpestris*	*flava*	M	19-Jun-06	61.42	8.87	KF735334	KF735259, KF735260	KF735513, KF735514
ZMUC123699	ZMUC 123699	*Eremophila alpestris*	*flava*			55.68	12.57	KF735504		
NHMUO8534	NHMUO 8534	*Eremophila alpestris*	*flava*		17-Jul-05	70.48	28.91	KF735343	KF735261, KF735262	
NHMUO30995	NHMUO 30995	*Eremophila alpestris*	*flava*	M	24-Jun-10	70.39	31.01	KF735340	KF735263, KF735264	KF735517, KF735518

Appendix S1. Samples used in this study and GenBank accession numbers

ID	Institution	Species	mtDNA clade	Sex	Date	Lat.	Lon.	ND2	ACO1	RHO
NHMUO8506	NHMUO 8506	*Eremophila alpestris*	*flava*		8-Jul-04	70.35	31.02	KF735342	KF735265	KF735523, KF735524
NHMUO31024	NHMUO 31024	*Eremophila alpestris*	*flava*	M	27-Jun-10	70.34	31.03	KF735341	KF735266, KF735267	KF735519, KF735520
MVK139	UWBM 74056	*Eremophila alpestris*	*flava*	F	18-May-01	59.83	52.72	KF735460	KF735268	KF735587, KF735588
MVK140	UWBM 74057	*Eremophila alpestris*	*flava*	F	18-May-01	59.83	52.72	KF735461		
MVK141	UWBM 74058	*Eremophila alpestris*	*flava*	F	18-May-01	59.83	52.72	KF735462		
SVD1431	UWBM 59593	*Eremophila alpestris*	*flava*	M	12-Jun-97	68.02	68.60	KF735470		
SVD1432	UWBM 59594	*Eremophila alpestris*	*flava*	F	12-Jun-97	68.02	68.60	KF735471		
SVD1433	UWBM 59595	*Eremophila alpestris*	*flava*	F	12-Jun-97	68.02	68.60	KF735472		
N85	MSUZM	*Eremophila alpestris*	*flava*		9-Aug-03	72.83	105.83	KF735463	KF735269	KF735589, KF735590
NAM173	UWBM 66344	*Eremophila alpestris*	*brandti*	M	19-Jun-00	50.38	89.87	KF735468		
MSUZM1792000	UWBM 75737	*Eremophila alpestris*	*brandti*	M	5-Jun-00	50.15	90.14	KF735451		
MSUZM1802000	UWBM 75738	*Eremophila alpestris*	*brandti*	M	5-Jun-00	50.15	90.14	KF735452		
MSUZM1812000	UWBM 75739	*Eremophila alpestris*	*brandti*	M	5-Jun-00	50.15	90.14	KF735453		
MSUZM1822000	UWBM 75740	*Eremophila alpestris*	*brandti*	M	5-Jun-00	50.15	90.14	KF735454		
MSUZM2242000	UWBM 75779	*Eremophila alpestris*	*brandti*	M	8-Jun-00	50.15	90.14	KF735455		
NAM161	UWBM 66332	*Eremophila alpestris*	*brandti*	F	17-Jun-00	50.35	90.48	KF735466		
NAM162	UWBM 66333	*Eremophila alpestris*	*brandti*	M	17-Jun-00	50.35	90.48	KF735467		
MSUZM1062000	UWBM 75668	*Eremophila alpestris*	*brandti*	M	27-May-00	50.62	91.52	KF735448		
MSUZM1242000	UWBM 75684	*Eremophila alpestris*	*brandti*	M	31-May-00	50.28	90.66	KF735449		
MSUZM1252000	UWBM 75685	*Eremophila alpestris*	*brandti*	M	31-May-00	50.28	90.66	KF735450		
NAM140	UWBM 66312	*Eremophila alpestris*	*brandti*	M	13-Jun-00	50.65	91.60	KF735464		
NAM142	UWBM 66314	*Eremophila alpestris*	*brandti*	F	13-Jun-00	50.65	91.60	KF735465		
MSUZMn0601999	UWBM 78960	*Eremophila alpestris*	*brandti*	M	29-May-99	51.35	94.55	KF735457		
MSUZMn0561999	UWBM 67579	*Eremophila alpestris*	*brandti*	F	28-May-99	51.42	94.77	KF735456		
MSUZMp0511999	UWBM 67679	*Eremophila alpestris*	*brandti*	M	6-Jun-99	50.03	95.03	KF735459		
RCF1976	UWBM 66603	*Eremophila alpestris*	*brandti*	M	9-Jul-00	50.07	95.13	KF735469		
MSUZMn2381999	UWBM 71026	*Eremophila alpestris*	*brandti*	M	29-Jun-99	50.28	96.40	KF735458		
DAB2299	UWBM 58019	*Eremophila alpestris*	*brandti*	M	11-Jun-97	48.12	100.37	KF735366		
B10281	USNM 586726	*Eremophila alpestris*	*brandti*		6-May-97	44.90	100.57	KF735347		
BKS3990	UWBM 57873	*Eremophila alpestris*	*brandti*	M	5-Jun-97	44.90	100.57	KF735353		
DAB2261	UWBM 57984	*Eremophila alpestris*	*brandti*	F	5-Jun-97	44.90	100.57	KF735364		
DAB2262	UWBM 57985	*Eremophila alpestris*	*brandti*	F	5-Jun-97	44.90	100.57	KF735365		
B10187	USNM 586670	*Eremophila alpestris*	*brandti*		28-May-97	43.36	103.18	KF735345		
B10275	USNM 586720	*Eremophila alpestris*	*brandti*		28-May-97	43.36	103.18	KF735346		
DAB2222	UWBM 57949	*Eremophila alpestris*	*brandti*	F	28-May-97	43.37	103.18	KF735362		
DAB2234	UWBM 57960	*Eremophila alpestris*	*brandti*	M	29-May-97	43.37	103.18	KF735363		

Appendix S1. Samples used in this study and GenBank accession numbers

ID	Institution	Species	mtDNA clade	Sex	Date	Lat.	Lon.	ND2	ACO1	RHO
LMC9745	AMNH	*Eremophila alpestris*	*brandti*		14-Jul-93	43.36	103.18	KF735439		
B10177	USNM 621499	*Eremophila alpestris*	*brandti*		24-May-97	47.33	105.40	KF735344		
CDS4894	UWBM 46334	*Eremophila alpestris*	*brandti*	F	16-Jun-93	51.57	106.85	KF735354	KF735270	KF735535, KF735536
CDS4895	UWBM 46335	*Eremophila alpestris*	*brandti*	M	16-Jun-93	51.57	106.85	KF735355	KF735271, KF735272	KF735537, KF735538
CDS4913	UWBM 46353	*Eremophila alpestris*	*brandti*	M	18-Jun-93	51.57	106.85	KF735356		
DAB2584	UWBM 60021	*Eremophila alpestris*	*brandti*	F	9-May-98	47.77	112.15	KF735367		
DAB2600	UWBM 60037	*Eremophila alpestris*	*brandti*	F	11-May-98	48.03	114.38	KF735368		
DAB2602	UWBM 60039	*Eremophila alpestris*	*brandti*	F	11-May-98	48.03	114.38	KF735369		
DAB2603	UWBM 60040	*Eremophila alpestris*	*brandti*	F	12-May-98	48.03	114.38	KF735370		
DAB2604	UWBM 60041	*Eremophila alpestris*	*brandti*	M	12-May-98	48.03	114.38	KF735371		
IUK2404	USNM 640019	*Eremophila alpestris*	*brandti*	F	28-Jun-05	50.50	115.00	KF735419		
IUK2405	USNM 640020	*Eremophila alpestris*	*brandti*	M	28-Jun-05	50.50	115.00	KF735420		
IUK2406	USNM 640021	*Eremophila alpestris*	*brandti*	F	28-Jun-05	50.50	115.00	KF735421		
IUK2413	YPM	*Eremophila alpestris*	*brandti*	F	28-Jun-05	50.50	115.00	KF735422		
IUK2414	USNM 640028	*Eremophila alpestris*	*brandti*	M	28-Jun-05	50.50	115.00	KF735423		
IUK2423	YPM	*Eremophila alpestris*	*brandti*	M	30-Jun-05	50.50	115.00	KF735424		
IUK2426	USNM 640037	*Eremophila alpestris*	*brandti*	M	30-Jun-05	50.50	115.00	KF735425		
IUK2427	USNM 640038	*Eremophila alpestris*	*brandti*	M	30-Jun-05	50.50	115.00	KF735426		
IVF0687	SDM	*Eremophila alpestris*	*brandti*	F	30-Jun-05	50.50	115.00	KF735427		
IVF0688	SDM	*Eremophila alpestris*	*brandti*	M	30-Jun-05	50.50	115.00	KF735428		
SVD3556	USNM 640648	*Eremophila alpestris*	*brandti*	M	28-Jun-05	50.50	115.00	KF735480	KF735273, KF735274	
SVD3568	USNM 640660	*Eremophila alpestris*	*brandti*	F	30-Jun-05	50.50	115.00	KF735481	KF735275	KF735613, KF735614
SVD3569	USNM 640661	*Eremophila alpestris*	*brandti*	M	30-Jun-05	50.50	115.00	KF735482	KF735276, KF735277	KF735615, KF735616
SVD3570	USNM 640662	*Eremophila alpestris*	*brandti*	M	30-Jun-05	50.50	115.00	KF735483	KF735278, KF735279	KF735617, KF735618
CSW5801	UWBM 59834	*Eremophila alpestris*	*brandti*	F	15-May-98	48.43	115.10	KF735357		
CSW5803	UWBM 59836	*Eremophila alpestris*	*brandti*	M	15-May-98	48.43	115.10	KF735358		
SVD2365	UWBM 66901	*Eremophila alpestris*	*alpestris*	M	2-Jun-00	65.73	194.14	DQ187408		
SVD2366	UWBM 66902	*Eremophila alpestris*	*alpestris*	M	2-Jun-00	65.66	193.82	DQ187409		
SVD2367	UWBM 66903	*Eremophila alpestris*	*alpestris*	M	3-Jun-00	65.97	193.82	DQ187410		
SVD2368	UWBM 66904	*Eremophila alpestris*	*alpestris*	F	3-Jun-00	65.97	193.82	DQ187411		
SVD2369	UWBM 66905	*Eremophila alpestris*	*alpestris*	M	6-Jun-00	65.43	195.23	DQ187412		
UAMX014	UAM 7608	*Eremophila alpestris*	*alpestris*	F	24-May-97	64.84	212.28	DQ187390		
B13413	USNM 622679	*Eremophila alpestris*	*alpestris*		22-Aug-00	64.58	213.25	DQ187391		
B13421	USNM 622678	*Eremophila alpestris*	*alpestris*		22-Aug-00	64.58	213.25	DQ187392		
B13422	USNM 622677	*Eremophila alpestris*	*alpestris*		22-Aug-00	64.58	213.25	DQ187393		
B13492	USNM 601741	*Eremophila alpestris*	*alpestris*		22-Aug-00	64.58	213.25	DQ187397		

Appendix S1. Samples used in this study and GenBank accession numbers

ID	Institution	Species	mtDNA clade	Sex	Date	Lat.	Lon.	ND2	ACO1	RHO
B13493	USNM 601742	*Eremophila alpestris*	*alpestris*		22-Aug-00	64.58	213.25	DQ187398		
B13425	USNM 622682	*Eremophila alpestris*	*alpestris*		22-Aug-00	64.63	213.39	DQ187394		
B13426	USNM 622681	*Eremophila alpestris*	*alpestris*		22-Aug-00	64.63	213.39	DQ187395		
B13485	USNM 601740	*Eremophila alpestris*	*alpestris*		22-Aug-00	64.64	213.40	DQ187396		
KSW1478	UAM 7752	*Eremophila alpestris*	*alpestris*	F	24-Jun-97	65.48	214.60	DQ187399		
KSW1479	UAM 7617	*Eremophila alpestris*	*alpestris*	M	24-Jun-97	65.48	214.60	DQ187400		
KSW1485	UAM 8957	*Eremophila alpestris*	*alpestris*	F	24-Jun-97	65.48	214.60	DQ187401		
KSW1500	UAM 7607	*Eremophila alpestris*	*alpestris*	F	25-Jun-97	65.48	214.60	DQ187402		
UAMX775	UAM 10087	*Eremophila alpestris*	*alpestris*	M	25-Jun-97	65.48	214.60	DQ187403		
UAMX790	UAM 10088	*Eremophila alpestris*	*alpestris*	M	25-Jun-97	65.48	214.60	DQ187404		
DAB0686	UWBM 53941	*Eremophila alpestris*	*alpestris*	M	22-Jun-95	65.50	214.62	DQ187405		
DAB0688	UWBM 53943	*Eremophila alpestris*	*alpestris*	F	22-Jun-95	65.50	214.62	DQ187406		
DAB0689	UWBM 53944	*Eremophila alpestris*	*alpestris*	F	22-Jun-95	65.50	214.62	DQ187407		
SVD3377	USNM 640482	*Eremophila alpestris*	*alpestris*	F	31-Jul-04	63.06	214.24			KF735611, KF735612
SVD4092	YPM	*Eremophila alpestris*	*alpestris*	M	6-Jul-07	64.59	218.73	KF735484	KF735280, KF735281	KF735619, KF735620
SVD4093	YPM	*Eremophila alpestris*	*alpestris*	M	6-Jul-07	64.59	218.73	KF735485	KF735282, KF735283	KF735621, KF735622
UBC28334	UBC28334	*Eremophila alpestris*	*alpestris*		nestling feather	54.78	232.72	KF735491		
UBC29857	UBC29857	*Eremophila alpestris*	*alpestris*		nestling feather	54.78	232.72	KF735492		
UBC29858	UBC29858	*Eremophila alpestris*	*alpestris*		nestling feather	54.78	232.72	KF735493		
UBC29860	UBC29860	*Eremophila alpestris*	*alpestris*		nestling feather	54.78	232.72	KF735494		
UBC29861	UBC29861	*Eremophila alpestris*	*alpestris*		nestling feather	54.78	232.72	KF735495		
UBC36201	UBC36201	*Eremophila alpestris*	*alpestris*		nestling feather	54.78	232.72	KF735496		
UBC36202	UBC36202	*Eremophila alpestris*	*alpestris*		nestling feather	54.78	232.72	KF735497		
UBC36206	UBC36206	*Eremophila alpestris*	*alpestris*		nestling feather	54.78	232.72	KF735498		
UBC36209	UBC36209	*Eremophila alpestris*	*alpestris*		nestling feather	54.78	232.72	KF735499		
UBC36218	UBC36218	*Eremophila alpestris*	*alpestris*		nestling feather	54.78	232.72	KF735500		
UBC36242	UBC36242	*Eremophila alpestris*	*alpestris*		nestling feather	54.78	232.72	KF735501	KF735284	KF735627, KF735628
SMB413	UWBM	*Eremophila alpestris*	*alpestris*			46.80	235.90	DQ187457	KF735285	KF735599, KF735600
SMB414	UWBM	*Eremophila alpestris*	*alpestris*			46.80	235.90	DQ187458		
SMB415	UWBM	*Eremophila alpestris*	*alpestris*			46.80	235.90	DQ187459		
SMB416	UWBM	*Eremophila alpestris*	*alpestris*			46.80	235.90	DQ187460		
SMB417	UWBM	*Eremophila alpestris*	*alpestris*			46.80	235.90	DQ187462		
SMB418	UWBM	*Eremophila alpestris*	*alpestris*			46.10	236.70	DQ187463		
SMB419	UWBM	*Eremophila alpestris*	*alpestris*			46.10	236.70	DQ187464		
SMB420	UWBM	*Eremophila alpestris*	*alpestris*			46.10	236.70	DQ187465		
SMB421	UWBM	*Eremophila alpestris*	*alpestris*			46.10	236.70	DQ187466		

Appendix S1. Samples used in this study and GenBank accession numbers

ID	Institution	Species	mtDNA clade	Sex	Date	Lat.	Lon.	ND2	ACO1	RHO
SMB422	UWBM	*Eremophila alpestris*	*alpestris*			46.10	236.70	DQ187467		
JMB758	UWBM 46848	*Eremophila alpestris*	*alpestris*	F	11-Aug-91	40.18	237.77	KF735438		
RCF2596	UWBM	*Eremophila alpestris*	*alpestris*			47.10	237.40	DQ187461		
SMB397	UWBM	*Eremophila alpestris*	*alpestris*			47.10	237.40	DQ187444		
SMB398	UWBM	*Eremophila alpestris*	*alpestris*			47.10	237.40	DQ187445		
SMB401	UWBM	*Eremophila alpestris*	*alpestris*			47.10	237.40	DQ187446		
SMB402	UWBM	*Eremophila alpestris*	*alpestris*			47.10	237.40	DQ187447		
SMB403	UWBM	*Eremophila alpestris*	*alpestris*			47.10	237.40	DQ187448		
SMB404	UWBM	*Eremophila alpestris*	*alpestris*			47.10	237.40	DQ187449		
SMB405	UWBM	*Eremophila alpestris*	*alpestris*			47.10	237.40	DQ187450		
SMB406	UWBM	*Eremophila alpestris*	*alpestris*			47.10	237.40	DQ187451		
SMB407	UWBM	*Eremophila alpestris*	*alpestris*			47.10	237.40	DQ187452		
SMB408	UWBM	*Eremophila alpestris*	*alpestris*			47.10	237.40	DQ187453		
SMB410	UWBM	*Eremophila alpestris*	*alpestris*			47.10	237.40	DQ187454		
SMB411	UWBM	*Eremophila alpestris*	*alpestris*			47.10	237.40	DQ187455		
SMB412	UWBM	*Eremophila alpestris*	*alpestris*			47.10	237.40	DQ187456		
EVL481	UWBM 77057	*Eremophila alpestris*	*alpestris*		21-May-02	47.09	237.42	DQ187437		
EVL483	UWBM 77059	*Eremophila alpestris*	*alpestris*		2-Jul-02	47.09	237.42	DQ187439		
EVL484	UWBM 77060	*Eremophila alpestris*	*alpestris*		2-Jul-02	47.09	237.42	DQ187440		
GKD375	UWBM 77062	*Eremophila alpestris*	*alpestris*		3-Jul-02	47.09	237.42	DQ187441		
GKD377	UWBM 77064	*Eremophila alpestris*	*alpestris*		17-Jul-02	47.09	237.42	DQ187443		
EVL482	UWBM 77058	*Eremophila alpestris*	*alpestris*		16-Jul-02	46.97	237.51	DQ187438		
GKD376	UWBM 77063	*Eremophila alpestris*	*alpestris*		25-Jun-02	46.97	237.51	DQ187442		
EVL485	UWBM 77061	*Eremophila alpestris*	*alpestris*	F	9-May-02	47.14	237.52	KF735395		
SVD0999	UWBM 53445	*Eremophila alpestris*	*alpestris*	M	19-Jul-95	48.77	238.08	DQ187484	KF735286, KF735287	KF735601, KF735602
SVD1000	UWBM 53410	*Eremophila alpestris*	*alpestris*	F	19-Jul-95	48.77	238.08	DQ187485		
SVD1001	UWBM 53411	*Eremophila alpestris*	*alpestris*	M	19-Jul-95	48.77	238.08	DQ187486		
NKJ5923	MVZ 171608	*Eremophila alpestris*	*alpestris*		30-Mar-88	37.63	238.53	DQ187413		
NKJ5924	MVZ 171609	*Eremophila alpestris*	*alpestris*		30-Mar-88	37.63	238.53	DQ187414		
NKJ5925	MVZ 171610	*Eremophila alpestris*	*alpestris*	M	30-Mar-88	37.63	238.53	DQ187415	KF735288, KF735289	
NKJ5926	MVZ 171611	*Eremophila alpestris*	*alpestris*	M	30-Mar-88	37.63	238.53	DQ187416	KF735290, KF735291	KF735591, KF735592
NKJ5927	MVZ 171612	*Eremophila alpestris*	*alpestris*		30-Mar-88	37.63	238.53	DQ187417		
NKJ5928	MVZ 171613	*Eremophila alpestris*	*alpestris*		30-Mar-88	37.63	238.53	DQ187418	KF735292, KF735293	
NKJ5929	MVZ 171614	*Eremophila alpestris*	*alpestris*		30-Mar-88	37.63	238.53	DQ187419		
NKJ5930	MVZ 171615	*Eremophila alpestris*	*alpestris*	F	30-Mar-88	37.63	238.53	DQ187420	KF735294	KF735593, KF735594
NKJ5931	MVZ 171616	*Eremophila alpestris*	*alpestris*		30-Mar-88	37.63	238.53	DQ187421		

Appendix S1. Samples used in this study and GenBank accession numbers

ID	Institution	Species	mtDNA clade	Sex	Date	Lat.	Lon.	ND2	ACO1	RHO
NKJ5932	MVZ 171617	*Eremophila alpestris*	*alpestris*		30-Mar-88	37.63	238.53	DQ187422		
NKJ5933	MVZ 171618	*Eremophila alpestris*	*alpestris*		30-Mar-88	37.63	238.53	DQ187423		
NKJ6003	MVZ 171619	*Eremophila alpestris*	*alpestris*		16-May-88	37.63	238.53	DQ187424		
NKJ6004	MVZ 171620	*Eremophila alpestris*	*alpestris*		16-May-88	37.63	238.53	DQ187425		
NKJ6005	MVZ 171621	*Eremophila alpestris*	*alpestris*		16-May-88	37.63	238.53	DQ187426		
NKJ6006	MVZ 171622	*Eremophila alpestris*	*alpestris*		16-May-88	37.63	238.53	DQ187427		
NKJ6007	MVZ 171623	*Eremophila alpestris*	*alpestris*		16-May-88	37.63	238.53	DQ187428		
NKJ6008	MVZ 171624	*Eremophila alpestris*	*alpestris*		16-May-88	37.63	238.53	DQ187429		
NKJ6009	MVZ 171625	*Eremophila alpestris*	*alpestris*		16-May-88	37.63	238.53	DQ187430		
NKJ6010	MVZ 171626	*Eremophila alpestris*	*alpestris*	F	16-May-88	37.63	238.53	DQ187431	KF735295	KF735595, KF735596
SVD2356	UWBM 66190	*Eremophila alpestris*	*alpestris*	M	16-Jun-00	42.48	239.72	DQ187432	KF735296, KF735297	KF735603, KF735604
SVD2357	UWBM 66191	*Eremophila alpestris*	*alpestris*	M	16-Jun-00	42.48	239.72	DQ187433		
SVD2358	UWBM 66194	*Eremophila alpestris*	*alpestris*	M	16-Jun-00	42.48	239.72	DQ187434		
SVD2359	UWBM 66192	*Eremophila alpestris*	*alpestris*	M	16-Jun-00	42.48	239.72	DQ187435		
SVD2360	UWBM 66193	*Eremophila alpestris*	*alpestris*	M	16-Jun-00	42.48	239.72	DQ187436		
SVD2207	UWBM 64947	*Eremophila alpestris*	*alpestris*	M	28-Apr-00	47.02	239.98	DQ187483		
SAR7267	UWBM 58576	*Eremophila alpestris*	*alpestris*	F	19-Jul-97	48.67	240.07	DQ187487		
DAB0411	UWBM 51057	*Eremophila alpestris*	*alpestris*	M	12-Mar-94	47.81	240.36	DQ187477		
CSW6422	UWBM 74049	*Eremophila alpestris*	*alpestris*	M	25-Apr-03	47.05	240.48	DQ187475		
CSW6423	UWBM 74050	*Eremophila alpestris*	*alpestris*	M	25-Apr-03	47.05	240.48	DQ187476		
CSW5717	UWBM 58529	*Eremophila alpestris*	*alpestris*	M	18-Jul-97	48.68	240.49	DQ187473		
CSW5718	UWBM 58530	*Eremophila alpestris*	*alpestris*		18-Jul-97	48.68	240.49	DQ187474		
PLG216	UWBM 58564	*Eremophila alpestris*	*alpestris*	M	18-Jul-97	48.68	240.49	DQ187478	KF735298, KF735299	KF735597, KF735598
SVD2197	UWBM 64941	*Eremophila alpestris*	*alpestris*	M	15-Apr-00	47.00	240.57	DQ187479		
SVD2198	UWBM 64942	*Eremophila alpestris*	*alpestris*	M	15-Apr-00	47.00	240.57	DQ187480		
SVD2199	UWBM 64943	*Eremophila alpestris*	*alpestris*	M	15-Apr-00	47.00	240.57	DQ187481		
SVD2200	UWBM 64944	*Eremophila alpestris*	*alpestris*	F	15-Apr-00	47.00	240.57	DQ187482		
CSW5140a	UWBM 52515	*Eremophila alpestris*	*alpestris*	F	18-Apr-95	47.61	240.71	DQ187471		
CSW5141a	UWBM 52516	*Eremophila alpestris*	*alpestris*	M	18-Apr-95	47.61	240.71	DQ187472		
BKS1405	UWBM 69686	*Eremophila alpestris*	*alpestris*	F	12-Mar-94	46.93	240.82	DQ187468		
BKS1417	UWBM 69698	*Eremophila alpestris*	*alpestris*	M	11-Mar-94	46.93	240.82	DQ187469		
BKS1419	UWBM 69700	*Eremophila alpestris*	*alpestris*	M	13-Mar-94	46.93	240.82	DQ187470		
JK00336	MBM 8470	*Eremophila alpestris*	*alpestris*		21-Jun-00	37.28	243.47	KF735431		
DHB3640	MBM 8472	*Eremophila alpestris*	*alpestris*		21-Jul-00	37.18	243.90	KF735378		
DHB3642	MBM 8474	*Eremophila alpestris*	*alpestris*		8-Jun-00	37.18	243.90	KF735379		
DHB3643	MBM 8473	*Eremophila alpestris*	*alpestris*		8-Jun-00	37.18	243.90	KF735380		

Appendix S1. Samples used in this study and GenBank accession numbers

ID	Institution	Species	mtDNA clade	Sex	Date	Lat.	Lon.	ND2	ACO1	RHO
DHB3644	MBM 8471	*Eremophila alpestris*	*alpestris*		8-Jun-00	37.18	243.90	KF735381		
DHB3646	MBM 8478	*Eremophila alpestris*	*alpestris*		8-Jun-00	37.18	243.90	KF735382		
DHB3647	MBM 8477	*Eremophila alpestris*	*alpestris*		8-Jun-00	37.18	243.90	KF735383		
DHB3648	MBM 8481	*Eremophila alpestris*	*alpestris*		8-Jun-00	37.18	243.90	KF735384		
DHB3649	MBM 8479	*Eremophila alpestris*	*alpestris*		8-Jun-00	37.08	243.90	KF735385		
DHB3650	MBM 8480	*Eremophila alpestris*	*alpestris*		8-Jun-00	37.08	243.90	KF735386		
DHB3982	MBM 9771	*Eremophila alpestris*	*alpestris*		22-May-01	37.18	243.92	KF735387		
DHB3992	MBM 9772	*Eremophila alpestris*	*alpestris*		22-May-01	37.18	243.92	KF735388		
DHB4005	MBM 9773	*Eremophila alpestris*	*alpestris*		22-May-01	37.18	243.92	KF735389		
JK00346	MBM 8504	*Eremophila alpestris*	*alpestris*		7-Jun-00	37.18	243.93	KF735432		
JK00348	MBM 8505	*Eremophila alpestris*	*alpestris*		7-Jun-00	37.18	243.93	KF735433		
GAV1938	MBM 8546	*Eremophila alpestris*	*alpestris*		12-Jul-00	37.12	243.95	KF735399		
GAV1939	MBM 8547	*Eremophila alpestris*	*alpestris*		12-Jul-00	37.12	243.95	KF735400		KF735561, KF735562
GAV1940	MBM 8548	*Eremophila alpestris*	*alpestris*		12-Jul-00	37.12	243.95	KF735401		
JK00354	MBM 8503	*Eremophila alpestris*	*alpestris*		12-Jun-00	37.12	243.95	KF735434		
GAV1937	MBM 8545	*Eremophila alpestris*	*alpestris*		7-Jun-00	37.12	244.00	KF735398		
B24753	LSUMNS	*Eremophila alpestris*	*alpestris*		7-May-96	33.13	244.49	KF735349		
DHB2538	MBM 5676	*Eremophila alpestris*	*alpestris*	M	11-Jul-98	37.68	246.94	KF735377	KF735300, KF735301	KF735541, KF735542
JDW0038	BMUM 43727	*Eremophila alpestris*	*alpestris*		18-Jun-94	46.60	247.05	KF735429		
B7308	USNM	*Eremophila alpestris*	*alpestris*			33.53	247.63	KF735352		
X7327	BMUM 43726	*Eremophila alpestris*	*alpestris*	M	19-Jun-94	48.51	249.03	KF735502		
JK9477	BMUM 43724	*Eremophila alpestris*	*alpestris*	M	20-Jun-94	48.57	249.70	KF735435		
JK9478	BMUM 43730	*Eremophila alpestris*	*alpestris*	M	20-Jun-94	48.57	249.70	KF735436		
JK9479	BMUM 43728	*Eremophila alpestris*	*alpestris*	F	20-Jun-94	48.57	249.70	KF735437		
X7328	BMUM 43725	*Eremophila alpestris*	*alpestris*	M	20-Jun-94	48.65	249.90	KF735503		
GAV0861	UWBM 56362	*Eremophila alpestris*	*alpestris*	M	24-Jun-96	38.69	253.52	KF735403	KF735302, KF735303	KF735559, KF735560
DHB1994	UWBM 70312	*Eremophila alpestris*	*alpestris*	F	21-Jun-96	39.60	254.29	KF735372		
DHB1995	UWBM 70313	*Eremophila alpestris*	*alpestris*	M	21-Jun-96	39.60	254.29	KF735373		
GAV0849	UWBM 56350	*Eremophila alpestris*	*alpestris*	F	22-Jun-96	39.79	254.23	KF735402		
CSW6291	UWBM 72563	*Eremophila alpestris*	*alpestris*	M	9-Jun-01	40.58	255.27	KF735359		KF735539, KF735540
CSW6292	UWBM 72564	*Eremophila alpestris*	*alpestris*	M	6-Jul-00	40.58	255.27	KF735360		
CSW6293	UWBM 72565	*Eremophila alpestris*	*alpestris*	M	22-Jun-00	40.58	255.27	KF735361		
EVL371	UWBM 72567	*Eremophila alpestris*	*alpestris*	M	20-May-00	40.58	255.27	KF735390		
EVL372	UWBM 72568	*Eremophila alpestris*	*alpestris*	M	5-Jun-01	40.58	255.27	KF735391		
EVL373	UWBM 72569	*Eremophila alpestris*	*alpestris*		9-Jul-01	40.70	255.22	KF735392		
EVL388	UWBM 72570	*Eremophila alpestris*	*alpestris*	M	18-May-01	40.58	255.27	KF735393		

Appendix S1. Samples used in this study and GenBank accession numbers

ID	Institution	Species	mtDNA clade	Sex	Date	Lat.	Lon.	ND2	ACO1	RHO
EVL389	UWBM 72571	*Eremophila alpestris*	*alpestris*	F	24-Jun-00	40.58	255.27	KF735394		
GKD200	UWBM 72572	*Eremophila alpestris*	*alpestris*	F	5-Jun-00	40.58	255.27	KF735404	KF735304	KF735563, KF735564
GKD201	UWBM 72573	*Eremophila alpestris*	*alpestris*	F	27-May-01	40.58	255.27	KF735405	KF735305	KF735565, KF735566
MLD042	UWBM 72574	*Eremophila alpestris*	*alpestris*	M	24-Jun-00	40.58	255.27	KF735440		
MLD043	UWBM 72575	*Eremophila alpestris*	*alpestris*	M	20-May-00	40.58	255.27	KF735441		
EVL766	UWBM 80629	*Eremophila alpestris*	*alpestris*	M	7-Jun-04	46.85	256.03	KF735396		
EVL769	UWBM 80632	*Eremophila alpestris*	*alpestris*	M	7-Jun-04	46.85	256.03	KF735397		
DHB2279	MBM 5161	*Eremophila alpestris*	*alpestris*	F	20-Jun-97	38.21	256.44	KF735374		
DHB2281	MBM 5155	*Eremophila alpestris*	*alpestris*	F	20-Jun-97	38.21	256.44	KF735375		
DHB2283	MBM 5154	*Eremophila alpestris*	*alpestris*	F	20-Jun-97	38.21	256.44	KF735376		
T208	UMMZ	*Eremophila alpestris*	*alpestris*		27-Jun-87	36.75	257.48	KF735490		
T1777	UMIMNH	*Eremophila alpestris*	*alpestris*		16-May-93	41.63	257.68	KF735489		
B2273	KUNHM	*Eremophila alpestris*	*alpestris*		28-Jun-00	38.17	263.59	KF735348		
JDW0097	BMUM 42513	*Eremophila alpestris*	*alpestris*	M	7-Mar-95	45.00	264.09	KF735430		
b3558	KUNHM	*Eremophila alpestris*	*alpestris*		15-Jun-00	39.98	264.81	KF735351		
b3548	KUNHM	*Eremophila alpestris*	*alpestris*		22-May-00	38.10	265.64	KF735350		
SVD2851	BMUM	*Eremophila alpestris*	*alpestris*		18-Oct-02	47.50	266.42	KF735477		
SVD2852	BMUM	*Eremophila alpestris*	*alpestris*		18-Oct-02	47.50	266.42	KF735478		
SVD2854	BMUM	*Eremophila alpestris*	*alpestris*			45.03	267.08	KF735479		
T1775	UMMZ	*Eremophila alpestris*	*alpestris*		11-Dec-93	45.56	275.32	KF735488		
HOLA12	UGA	*Eremophila alpestris*	*alpestris*		egg shell	33.96	276.62	KF735406		KF735567, KF735568
HOLA15	UGA	*Eremophila alpestris*	*alpestris*		egg shell	33.96	276.62	KF735407		
HOLA17	UGA	*Eremophila alpestris*	*alpestris*		egg shell	33.96	276.62	KF735408		
HOLA19	UGA	*Eremophila alpestris*	*alpestris*		egg shell	33.96	276.62	KF735409		
HOLA22	UGA	*Eremophila alpestris*	*alpestris*		egg shell	33.96	276.62	KF735410		
HOLA26	UGA	*Eremophila alpestris*	*alpestris*		egg shell	33.96	276.62	KF735411		KF735569, KF735570
HOLA32	UGA	*Eremophila alpestris*	*alpestris*		egg shell	33.96	276.62	KF735412		
HOLA33	UGA	*Eremophila alpestris*	*alpestris*		egg shell	33.96	276.62	KF735413		
HOLA40	UGA	*Eremophila alpestris*	*alpestris*		egg shell	33.96	276.62	KF735414		
HOLA52	UGA	*Eremophila alpestris*	*alpestris*		egg shell	33.96	276.62	KF735415		
HOLA72	UGA	*Eremophila alpestris*	*alpestris*		egg shell	33.96	276.62	KF735416		
HOLA78	UGA	*Eremophila alpestris*	*alpestris*		egg shell	33.96	276.62	KF735417		
HOLA97	UGA	*Eremophila alpestris*	*alpestris*		egg shell	33.96	276.62	KF735418		
1B446	ROM	*Eremophila alpestris*	*alpestris*			43.65	280.62	KF735335		
1B592	ROM	*Eremophila alpestris*	*alpestris*			43.65	280.62	KF735336		
1B593	ROM	*Eremophila alpestris*	*alpestris*			43.65	280.62	KF735337		

Appendix S1. Samples used in this study and GenBank accession numbers

ID	Institution	Species	mtDNA clade	Sex	Date	Lat.	Lon.	ND2	ACO1	RHO
1B769	ROM	*Eremophila alpestris*	*alpestris*			43.65	280.62	KF735338		
1B771	ROM	*Eremophila alpestris*	*alpestris*	M		43.65	280.62	KF735339	KF735306, KF735307	KF735515, KF735516
SVD2384	UWBM 68259	*Eremophila alpestris*	*alpestris*	M	15-Mar-92	42.67	283.46	KF735476	KF735308, KF735309	KF735609, KF735610
SVD2361	UWBM 68254	*Eremophila alpestris*	*alpestris*	F	16-Aug-00	46.66	306.93	KF735473	KF735310	KF735605, KF735606
SVD2362	UWBM 68255	*Eremophila alpestris*	*alpestris*	F	16-Aug-00	46.66	306.93	KF735474		KF735607, KF735608
SVD2363	UWBM 68253	*Eremophila alpestris*	*alpestris*		16-Aug-00	46.66	306.93	KF735475		
